# Identification of commonly altered genes between in major depressive disorder and a mouse model of depression

**DOI:** 10.1038/s41598-017-03291-x

**Published:** 2017-06-08

**Authors:** Hirotaka Yamagata, Shusaku Uchida, Koji Matsuo, Kenichiro Harada, Ayumi Kobayashi, Mami Nakashima, Masayuki Nakano, Koji Otsuki, Naoko Abe-Higuchi, Fumihiro Higuchi, Toshio Watanuki, Toshio Matsubara, Shigeo Miyata, Masato Fukuda, Masahiko Mikuni, Yoshifumi Watanabe

**Affiliations:** 1 Division of Neuropsychiatry, Department of Neuroscience, Yamaguchi University Graduate School of Medicine, 1-1-1 Minami-kogushi, Ube, Yamaguchi 755-8505 Japan; 2 Nagatoichinomiya Hospital, 17-35 Katachiyama-midoricho, Shimonoseki, Yamaguchi 751-0885 Japan; 3 Katakura Hospital, 229-3 Nishikiwa, Ube, Yamaguchi 755-0151 Japan; 4 Department of Psychiatry, Shimane University Faculty of Medicine, 89-1 Enya-cho, Izumo, Shimane 693-8501 Japan; 5 Health Service Center Organization for University Education, Yamaguchi University, 1677-1 Yoshida, Yamaguchi-shi, Yamaguchi 753-8511 Japan; 6 Departments of Psychiatry and Neuroscience, Gunma University Graduate School of Medicine, 3-39-22 Showa-machi, Maebashi, Gunma 371-8511 Japan; 7 Hakodate Watanabe Hospital, 1-31-1 Yunokawa-cho, Hakodate, Hokkaido 042-8678 Japan; 8 Department of Psychiatry, Hokkaido University Graduate School of Medicine, North 15, West 7, Kita-Ku, Sapporo, Hokkaido 060-8638 Japan

## Abstract

The heterogeneity of depression (due to factors such as varying age of onset) may explain why biological markers of major depressive disorder (MDD) remain uncertain. We aimed to identify gene expression markers of MDD in leukocytes using microarray analysis. We analyzed gene expression profiles of patients with MDD (age ≥50, age of depression onset <50) (N = 10, depressed state; N = 13, remitted state). Seven-hundred and ninety-seven genes (558 upregulated, 239 downregulated when compared to those of 30 healthy subjects) were identified as potential markers for MDD. These genes were then cross-matched to microarray data obtained from a mouse model of depression (676 genes, 148 upregulated, 528 downregulated). Of the six common genes identified between patients and mice, five genes (SLC35A3, HIST1H2AL, YEATS4, ERLIN2, and PLPP5) were confirmed to be downregulated in patients with MDD by quantitative real-time polymerase chain reaction. Of these genes, HIST1H2AL was significantly decreased in a second set of independent subjects (age ≥20, age of onset <50) (N = 18, subjects with MDD in a depressed state; N = 19, healthy control participants). Taken together, our findings suggest that HIST1H2AL may be a biological marker of MDD.

## Introduction

Depression is one of the most prevalent mental disorders found in ageing populations, and is associated with an increase in mortality, disability, and healthcare costs^1^. Accurate diagnosis of depression is important for early intervention in primary care^2^. However, because symptoms of geriatric depression are often atypical, it is difficult to diagnose depression in elderly individuals using operational diagnostic criteria (International Classification of Diseases, 10^th^ revision or Diagnostic and Statistical Manual of Mental Disorders, fifth edition; DSM-5). Moreover, the diagnosis of depression in elderly patients is often confounded by the presence of several somatic comorbidities^3^. Thus, biological markers of depression are required for primary care of geriatric patients.

Many biological markers of depression have been reported. Such markers include regional differences in specific brain areas (visualized using imaging techniques)^4^, as well as alterations in cytokine expression patterns^5^, neurotrophic factors^6^, and transcriptomes of peripheral leukocytes^7^. We previously reported reduced levels of glial cell line-derived neurotrophic factor mRNA in patients with major depressive disorder (MDD) when compared to healthy controls^8^. We also reported alterations in the mRNA levels of various histone deacetylases^9^, sirtuins^10^, and DNA methyltransferases^11^, as well as the target genes of hypoxia-inducible factor 1^12^, in patients with MDD. Although the above observations have not yet been of practical use in the diagnosis of depression, they have aided our understanding of the pathophysiology of MDD^13, 14^. Recently, we identified potential biomarkers of late-onset MDD in the transcriptomes of human leukocytes, which we were then able to successfully cross-match to an animal model of depression^15^. Such biomarkers could be used for the diagnosis of late-onset MDD, and may additionally provide clues helpful in elucidating the pathophysiology of this disorder.

It has been suggested that depression is a heterogeneous disease, both in its etiology and pathophysiology. Variability in the age of onset of depression contributes to the heterogeneous nature of this disorder^16^. Genetic factors have been implicated in adult-onset MDD rather than in late-onset MDD. On the other hand, identifying specific markers of MDD might be difficult because the diagnoses for 10–20% of patients with MDD will be changed from MDD to bipolar disorder over time^17^. One clinical study also indicated that about 40% of patients with MDD have hypomanic symptoms^18^. Studying patients with MDD without manic episodes until old age will provide clinicians with clues regarding how they can rule out a diagnosis of bipolar disorder in patients with depression. However, few clinical studies have been conducted on patients with adult-onset MDD who have never experienced manic episodes until old age.

In this study, we used microarray analysis to search for gene expression markers of confirmed MDD with onset before the age of 50 and without manic episode until the age of 50 and older in the leukocytes. Identified genes were then compared to the microarray data of mRNA obtained from the blood of chronic, ultra-mildly stressed (CUMS) BALB/c mice which served as an animal model of depression^13^. Finally, we investigated whether our identified markers were altered in young patients (≥20 years of age) with MDD.

## Results

Demographic and clinical data from the participants in Experiment 1 (aged ≥50 years, age of depression onset <50 years) are shown in Table 1. Participants with MDD who were in a depressed state (DP) were younger than those in the healthy control (HC) group. Additionally, participants with MDD who were in DP had greater imipramine-equivalent doses than those in a remitted state (RM). In total, 5,156 candidate probes were selected based on significant expression differences among the HC (N = 30), MDD-DP (N = 10), and MDD-RM (N = 13) groups, as determined using a one-way analysis of variance (ANOVA) with the false discovery rate (FDR). Of these probes, 797 (558 upregulated, 239 downregulated) with annotated gene symbols exhibited absolute fold-changes that were larger than 1.3 and that were significantly different between the HC and MDD-DP groups, as determined by a post hoc analysis. The top 50 probes are shown in Table S1.

**Table 1:** Demographic data of participants in Experiment 1.

	HC	MDD-DP	MDD-RM
No. of subjects	30	10	13
Sex (male/female)	13/17	5/5	8/5
Age (years)	63.3 ± 8.0	56.0 ± 6.8	57.7 ± 7.7
Age at onset (years)	—	35.7 ± 8.9	41.0 ± 8.8
Duration of illness (years)	—	20.3 ± 7.9	16.7 ± 12.8
HRDS	0.5 ± 0.9	21.7 ± 2.2^*#^	5.1 ± 2.7^*^
Equivalent dose of imipramine (mg)	—	221 ± 131^#^	90 ± 59

Demographic data of healthy controls (HCs) and patients with major depressive disorder in depressed (MDD-DP) and remitted (MDD-RM) states. Data indicate the mean ± SD. *p < 0.05 DP vs. HC, ^#^p < 0.05 MDD-DP vs. MDD-RM.

**Figure 1:**
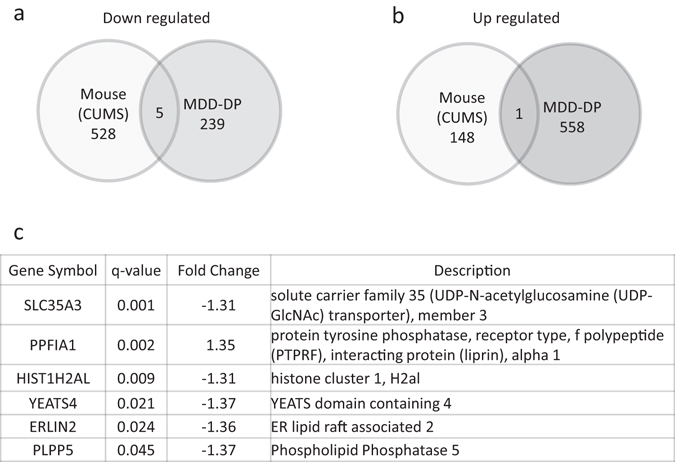
Venn diagrams of significantly altered genes in patients with MDD-DP and in a mouse model of depression. (**a**) Comparison of significantly upregulated genes. (**b**) Comparison of significantly downregulated genes. (**c**) List of common candidate genes in blood from patients with MDD-DP and a mouse model of depression.

We investigated whether altered gene expression in the MDD-DP group could be observed in a mouse model of depression. We previously reported that BALB/c mice are susceptible to CUMS^13, 14, 19^. In addition, imipramine treatment prevents BALB/c mice from exhibiting increased depression-like behaviors after CUMS exposure^13, 19^. We analyzed mRNA expression in blood from mice in the CUMS^13^ (CUMS, N = 4), CUMS and imipramine treatment (IMI, N = 4), and non-stressed (NS, N = 4) groups using a one-way ANOVA (p-value < 0.05). mRNA levels were analyzed without FDR because of the small numbers of mice in each group. Of the selected probes (1,032 probes), 676 probes (148 upregulated, 528 downregulated) with annotated gene symbols exhibited absolute fold-changes larger than 1.3 between the CUMS and NS groups. The top 50 probes are shown in Table S2. We next cross-matched the genes that were altered in patients with MDD-DP to those altered in animals in the CUMS group, and identified six candidate genes (*SLC35A3*, *PPFIA1*, *HIST1H2AL*, *YEATS4*, *ERLIN2*, and *PLPP5*) (Fig. 1a–c).

**Figure 2:**
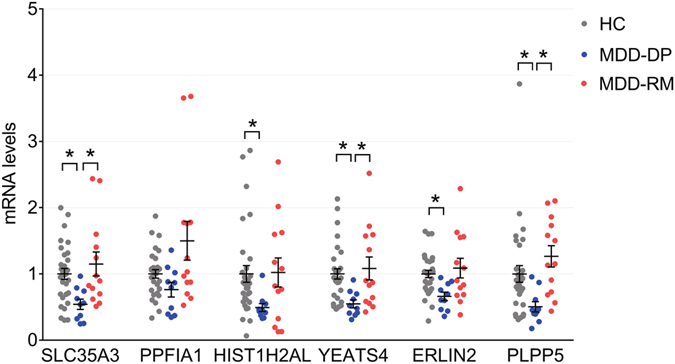
The expression levels of candidate genes in human leukocytes (participants from Experiment 1). Expression levels as dots with average line ± SEM for *SLC35A3*, *PPFIA1*, *HIST1H2AL*, *YEATS4*, *ERLIN2*, and *PLPP5* mRNA in the leukocytes of patients with MDD-DP and HCs (Experiment 1). *p < 0.05.

We performed real-time quantitative real-time polymerase chain reaction (Q-PCR) to validate changes in the expression levels of these genes in patients with MDD-DP in Experiment 1. The expression levels of five genes (*SLC35A3*, *HIST1H2AL*, *YEATS4*, *ERLIN2*, and *PLPP5*) were significantly lower in the MDD-DP group than in the HC group (Fig. 2). As previously mentioned, participants in the MDD-DP group were younger than those in the HC group. However, none of the altered expression levels correlated with age (correlation coefficient and p-value: *SLC35A3*, 0.01, p = 0.94, *HIST1H2AL*; −0.05, p = 0.73, *YEATS4*; 0.147, p = 0.29, *ERLIN2*; 0.11, p = 0.446; *PLPP5*, 0.010, p = 0.94). In contrast, *SLC35A3*, *YEATS4*, *ERLIN2*, and *PLPP5* expression levels correlated with the imipramine-equivalent dose (correlation coefficient and p value: *SLC35A3*, −0.427, p = 0.042; *HIST1H2AL*, −0.228, p = 0.30; *YEATS4*, −0.448, p = 0.032; *ERLIN2*, −0.475, p = 0.022; *PLPP5*, −0.577, p = 0.004). The expression levels of candidate genes did not correlate with the duration of illness (correlation coefficient and p value: *SLC35A3*, −0.026, p = 0.91; *HIST1H2AL*, −0.224, p = 0.31; *YEATS4*, −0.213, p = 0.33; *ERLIN2*, −0.0746, p = 0.74; *PLPP5*, −0.011, p = 0.96). The areas under the ROC curves (AUCs) for the MDD-DP group were 0.813 (95% confidence interval [CI]: 0.678–0.949), 0.813 (95% CI: 0.657–0.970), 0.840 (95% CI: 0.706–0.974), 0.797 (95% CI: 0.658–0.935), and 0.767 (95% CI: 0.609–0.924) for *SLC35A3*, *HIST1H2AL*, *YEATS4*, *ERLIN2*, and *PLPP5*, respectively (Fig. S1).

**Figure 3:**
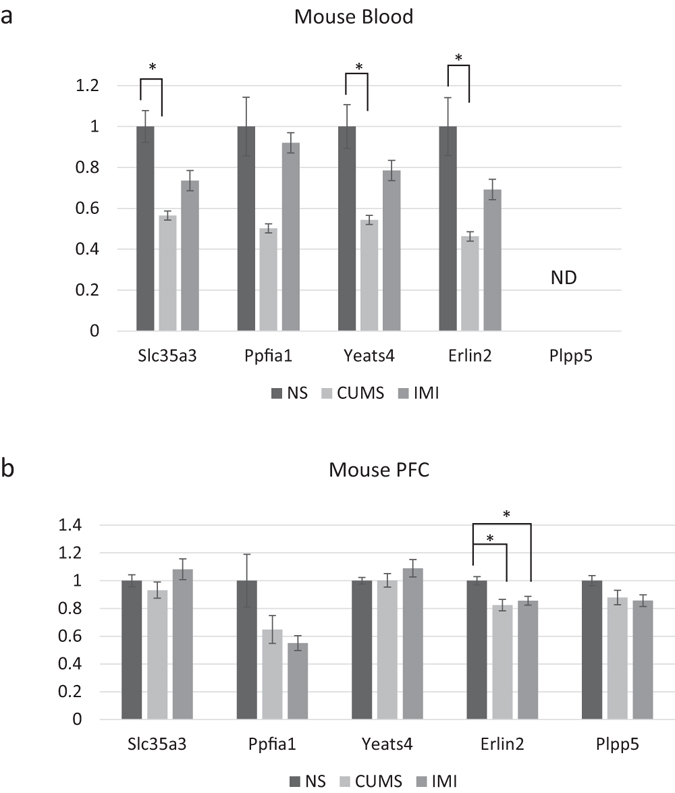
The expression levels of candidate genes in murine blood cells, and in murine prefrontal cortex. (**a**,**b**) The expression levels of *Slc35a3*, *Ppfia1*, *Yeats4*, *Erlin2*, and *Plpp5* mRNA in non-stressed (NS), chronic, ultra-mildly stressed (CUMS), and CUMS + imipramine-treated mice (IMI) (**a**; blood cells; NS, N = 6; CUMS, N = 6; IMI, N = 5) (**b**; PFC; NS, N = 8; CUMS, N = 8; IMI, N = 8). Specific primers for *Hist1h2al* could not be designed. *Plpp5* could not be detected (ND). Data indicate the mean ± SEM. *p < 0.05.

In mouse blood, three genes (*Slc35a3*, *Yeats4*, and *Erlin2*) were significantly decreased by CUMS, as determined by real-time Q-PCR (Fig. 3a). Unfortunately, we could not design specific and suitable primers for *Hist1h2al* for real-time Q-PCR. Moreover, *Plpp5* could not be detected in the blood. Interestingly, *SLC35A3*, *YEATS4*, and *ERLIN2* were also found to be altered in the blood of patients with MDD-DP. Next, we investigated gene expression in the hippocampus and prefrontal cortex (PFC) of mice following CUMS and imipramine treatment. In the PFC, the expression of *Erlin2* was significantly decreased in mice in the CUMS and IMI groups (Fig. 3b). In contrast, only *Erlin2* was decreased in the hippocampus of mice in the IMI group (Fig. S2).

**Figure 4:**
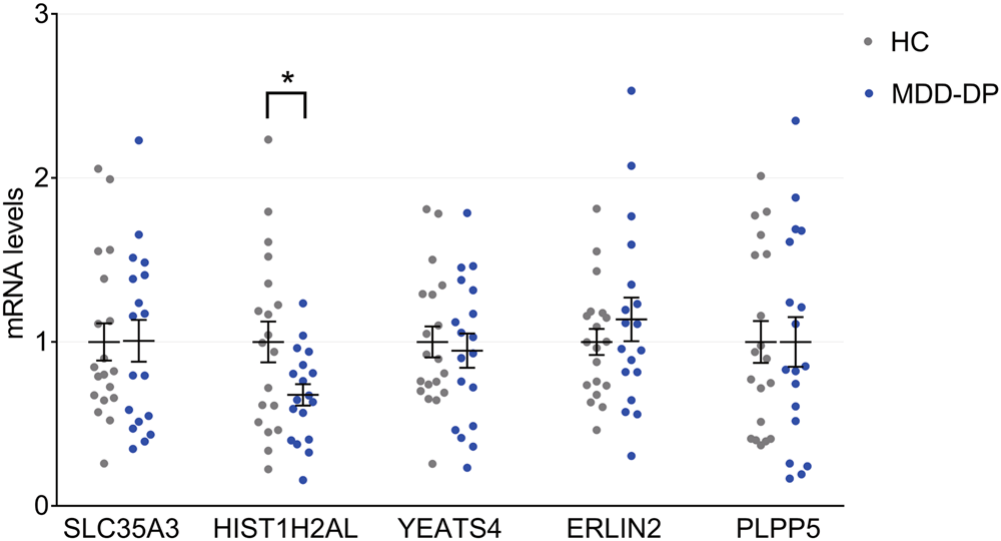
The expression levels of candidate genes in human leukocytes (participants from Experiment 2). The expression levels as dots with average line ± SEM for *SLC35A3*, *PPFIA1*, *HIST1H2AL*, *YEATS4*, *ERLIN2*, and *PLPP5* mRNA in leukocytes from patients with MDD-DP and HCs (Experiment 2). *p < 0.05.

In Experiment 2 (aged ≥20 years, age of depression onset <50 years), we investigated whether the abovementioned genes are reliable markers of MDD-DP using real-time Q-PCR (Fig. 4). For this experiment, participants in the MDD group included young patients (demographic and clinical data are shown Table 2). The expression of *HIST1H2AL* was decreased in patients with MDD-DP when compared to HCs. However, *SLC35A3*, *YEATS4*, *ERLIN2*, and *PLPP5* expression profiles were not different between the two groups. Interestingly, the expression levels of the latter four genes were correlated with age in younger participants (correlation coefficient and p value: *SLC35A3*, −0.439, p = 0.007; *HIST1H2AL*, −0.256, p = 0.13; *YEATS4*, −0.425, p = 0.009; *ERLIN2*, −0.495, p = 0.002; *PLPP5*, −0.457, p = 0.004). *SLC35A3* and *PLPP5* were found to be correlated with imipramine-equivalent dose (correlation coefficient and p value: *SLC35A3*, 0.505, p = 0.033; *HIST1H2AL*, 0.101, p = 0.69; *YEATS4*, 0.289, p = 0.25; *ERLIN2*, 0.276, p = 0.27; *PLPP5*, 0.719, p = 0.0008). The expression levels of candidate genes did not correlate with disease duration (correlation coefficient and p value: *SLC35A3*, −0.102, p = 0.69; *HIST1H2AL*, −0.151, p = 0.55; *YEATS4*, −0.098, p = 0.70; *ERLIN2*, −0.084, p = 0.74; *PLPP5*, −0.262, p = 0.29).

**Table 2:** Demographic data of participants in Experiment 2.

	HC	MDD-DP
No. of subjects	19	18
Sex (male/female)	8/11	7/11
Age (years)	38.3 ± 12.7	41.9 ± 11.2
Age at onset (years)	—	30.9 ± 9.6
Duration of illness (years)	—	11.1 ± 8.6
HDRS	0.7 ± 1.1	21.7 ± 2.9*
Equivalent dose of imipramine (mg)	—	250 ± 153

Demographic data of HCs and patients with MDD-DP. Data indicate the mean ± SD. *p < 0.05 MDD-DP vs. HC.

## Discussion

Blood biomarkers have proven to be both useful and minimally invasive in the detection and diagnosis of disease. In the current study, we identified candidate MDD biomarkers in the blood transcriptomes of middle-aged and older patients. Specifically, we found that *HIST1H2AL*, *ERLIN2*, *SLC35A3*, *YEATS4*, and *PLPP5* could all be biomarkers for middle-aged and older patients with MDD-DP, and that *HIST1H2AL* expression was significantly altered in independent samples from younger patients with MDD-DP. Together, our findings suggest that these genes may be useful for the assessment of MDD-DP and the elucidation of its pathophysiology.

Several previous groups have reported different transcriptomic blood markers for the detection of depression^20–25^. However, a valid and reliable biomarker has not yet been identified. One reason for this is the heterogeneity of “major depression”, which may include both bipolar^18, 26^ and adjustment disorders^27^. In the current study, we sought to discover a rigid genetic marker of MDD, and aimed to circumvent the heterogeneity of depressed populations by selecting patients with MDD who had not experienced a manic episode until the age of 50 years or older. Consequently, study participants in Experiment 1 comprised patients with MDD-DP who were classified as having recurrent depressive disorder only. We then cross-matched candidate genes discovered in our patient population to those from a mouse model of depression.

*HIST1H2AL* encodes one of the core histone proteins, Histone H2A type 1^28^. Histone variants regulate gene expression through changes in nucleosome composition and histone modification^29^. Moreover, histone variants are important for neurodevelopment^30^, and epigenetic histone modification plays a crucial role in depression^31^. We previously reported altered expression levels of histone deacetylases, including sirtuins and DNA methyltransferases, in patients with MDD^9–11^. However, little is known about how histone variants are affected by depression. In the current study, the top 50 genes found using microarray data from patients with MDD-DP included three histone variants: *HIST1H2AI*, *HIST1H4A*, and *HIST1H4L* (Table S1). These three genes and *HIST1H2AL* are located on chromosome 6p22.1–6p22.2, which has a histone gene cluster and a region containing immunity-related genes^32^. Chromosome 6p is reportedly related to bipolar disorder susceptibility^33^ and schizophrenia, as determined in a genome-wide association study (GWAS)^32^. Another GWAS study suggested that there are common neural, immune, and histone pathways in schizophrenia, bipolar disorder, and MDD^34^. In addition, we observed that *HIST1H2AL* was the only gene that was altered in both middle-aged and older patients (Experiment 1) and young patients (Experiment 2) with MDD-DP. Together with the results of previous reports, these findings suggest that *HIST1H2AL* on chromosome 6p22.1 is a marker of MDD.

In this study, *ERLIN2*, *SLC35A3*, *and YEATS4*, which are described in detail in the following paragraphs, were significantly decreased both in leukocytes from patients with MDD-DP, and in the blood from CUMS mice. We recently reported elevated levels of cell death-inducing DFFA-like effector c (CIDEC), solute carrier family 36 member-1 (SLC36A1), ribonuclease 1 (RNASE1), and serine/threonine/tyrosine interacting-like 1 (STYXL1) in patients with late-onset MDD and in ovariectomized mice exposed to CUMS, which is an animal model of postmenopausal depression^15^. Importantly, these MDD marker genes identified in this study did not overlap with previously reported late-onset MDD marker genes. Using methods similar to those used in our study, Pajer and colleagues reported common blood transcriptomic markers between patients with early-onset depression and a rat model of depression^20^. Of note, the genes discovered by Pajer *et al*. were different from the ones reported here. However, subjects in the previous study were much younger (age, 16.6 ± 1.3) than the participants described here (56.0 ± 6.8). In addition, the animal model used in the above study (i.e., a Fischer 344 rat) had chronic restraint stress^35^, while we used BALB/c mice subjected to variable forms of chronic stress^13^.

*ERLIN2* (endoplasmic reticulum lipid raft-associated 2) is a lipid raft-associated protein^36^ that blocks the polyubiquitination and degradation of inositol 1,4,5-triphosphate receptors (IP3Rs)^37^. IP3Rs have multiple functions in cells throughout the body, including neurons^38^. It has been shown that IP3R knockdown and blockade exert antidepressant effects^39^. Moreover, ERLIN2 knockdown suppresses the growth of cultured neurons, while ERLIN2 mutations lead to juvenile primary lateral sclerosis^40^. ERLIN2 has also been shown to modulate endoplasmic reticulum-related stress pathways^41^. Here, we found decreased expression levels of ERLIN2 in the PFC of stressed mice, which, based on previous literature, could promote IP3R degradation and neuron loss in the brain. Future studies on ERLIN2 function using various techniques (e.g., genetically engineered mice) will help elucidate the role of this gene in depression.

*SLC35A3* (solute carrier family 35 member A3) is a uridine diphosphate N-acetylglucosamine transporter^42^. Mutations in *SLC35A3* lead to vertebral malformations, autism spectrum disorder, epilepsy, and arthrogryposis^43, 44^. It was recently reported that *SLC35A3* interacts with mannoside acetylglucosaminyltransferases (Mgats)^45^, and that Mgat5−/− mice exhibit decreased depressive behavior and resistance to chronic stress^46^. Thus, *SLC35A3* may be associated with depression via its actions on Mgat5.

*YEATS4* (YEATS domain containing 4), or GAS41, is a transcription factor that is amplified in human gliomas^47^ and is a negative regulator of the p53 pathway^48, 49^. We also identified *TP53*, which encodes p53, as an altered gene in patients with MDD-DP (Table S1). Several reports have suggested that apoptotic factors may be related to mood disorders, including major depression^50^. However, it remains unclear how *YEATS4* is associated with depressive disorder.

Finally, *PLPP5* (phospholipid phosphatase 5) contains an acid phosphatase homologue domain^51^ and has been reported to be associated with some cancers, including hepatocyte carcinoma and breast cancer^51, 52^. It has been reported that *PLPP5* regulates protein kinase C, Janus-activated kinase-signal transducer and activator of transcription, and mitogen-activated protein kinase pathways^52^. While these pathway have all been associated with mood disorders^53, 54^, how *PLPP5* relates to MDD is not well understood. Thus, additional research is required to confirm the functions of *PLPP5* in stress and MDD.

This study had some limitations that should be addressed. First, our study sample was small because of the small number of recruited middle-aged and older patients with MDD. Second, we were unable to exclude an effect of aging, because while we showed that *SLC35A3*, *YEATS4*, *ERLIN2*, and *PLPP5* expression levels did not significantly correlate with age in patients over 50 years of age (Experiment 1), the expression levels of these genes did correlate with age in younger participants (Experiment 2). However, it should be noted that these genes were not significantly altered in patients that participated in the second set of experiments. Third, the patients in Experiment 1 were diagnosed with MDD without a manic episode until the age of 50 and over. However, the patients in Experiment 2 were younger than those in Experiment 1, and might be diagnosed with bipolar disorder in the future. This difference might affect the expression levels of some genes. Fourth, most of the patients were on antidepressants at the time of the study, and *SLC35A3*, *YEATS4*, *ERLIN2*, and *PLPP5* were found to correlate with the imipramine-equivalent dose. Thus, we cannot exclude the possibility that antidepressants might decrease the expression levels of these genes. However, *Slc35a3*, *Yeats4*, and *Erlin2* were significantly decreased in murine blood after CUMS, and importantly, in the absence of antidepressant treatment. This suggests that antidepressants do not affect the expression levels of the above genes. Fifth, because of the high homology between *Hist1h2al* and other histone variants (e.g., *Hist2h2aa1*), we could not quantify murine *Hist1h2al* using real-time Q-PCR, as we were unable to produce specific primers for this gene. Sixth, we could not exclude the random probability of selecting common genes between the patients and the mice. However, the candidate genes were validated using real-time PCR. Other important genes might have been missed during the process of cross-matching. Finally, the function of the vast majority of these genes in the brain remains unclear and requires further investigation.

In conclusion, we used a genome-wide microarray analysis of gene expression to identify candidate genes that were found both in the blood of patients with MDD and in a mouse model of depression. *HIST1H2AL* in particular may prove useful as a diagnostic marker of MDD.

## Methods

### Subjects

#### Participants in Experiment 1 (aged ≥50 years, age of depression onset <50 years)

The Institutional Review Board of Yamaguchi University Hospital approved this study and all subjects provided written informed consent for participation. This study was carried out in accordance with the latest version of the Declaration of Helsinki.

Middle-aged and older patients (aged ≥50 years) and healthy participants were recruited according to a previous report^15, 55^. Briefly, patients were recruited from Yamaguchi University Hospital and referred by clinics and hospitals in the area, while healthy control participants (HC) were recruited using advertisements in the community. Patients were diagnosed with depression using a clinical interview and the International Neuropsychiatric Interview (M.I.N.I., Japanese version 5.0.0)^56^. A depressed state was defined as a score greater than 18 on the Hamilton Rating for Depression Scale (HRDS)^57^. Patients in a remitted state met the DSM-IV criteria for full remission. With the exception of two patients (with HRDS scores of 8), most of the remitted patients had scores of less than 8 on the HRDS. Twenty-one participants met the criteria for MDD (age of depression onset <50 years). Two of the 21 patients were classified as being in both depressed and remitted states. We therefore had 21 patients in the study, although the total number of samples was 23. Ten samples were collected from patients in a depressed state (MDD-DP) while 13 samples were collected from patients in a remitted state (MDD-RM). HC participants were screened using the M.I.N.I. and the clinical interview. Any HC participant who had an immediate family member with any psychiatric disorder was excluded. Exclusion criteria for patients included current or past history of substance abuse/dependence, other psychotic illnesses, endocrine disease, head trauma, neurological disease, family history of hereditary neurological disorder, or other severe medical conditions (e.g., liver failure and kidney failure). All subjects received a full explanation of the study and provided written informed consent before agreeing to participate in the study. Demographic data are summarized in Table 1. Blood samples (collected between 2012 and 2013) obtained from MDD-DP (N = 10), MDD-RM (N = 13), and HC (N = 30) participants were analyzed using a gene expression assay.

#### Participants in Experiment 2 (aged ≥20 years, age of depression onset <50 years)

The Institutional Review Board of Yamaguchi University Hospital approved this study and all subjects provided written informed consent for participation. This study was carried out in accordance with the latest version of the Declaration of Helsinki.

Patients with MDD and healthy participants were recruited according to the protocol used for subject enrollment in Experiment 1. In order to investigate whether the markers identified in Experiment 1 were useful in detecting MDD in young patients, we performed our second experiment on both participants aged ≥50 years and those aged 20–49 years. Blood samples (collected between 2013 and 2015) from both MDD-DP (N = 18) and age- and sex-matched HC participants (N = 19) were analyzed using a gene expression assay. Demographic data are summarized in Table 2.

### RNA isolation from human blood

Total RNA isolation from blood samples was performed according to previously reported methods^8, 58^, with some minor revisions. In brief, venous blood was collected from the participants between 9:00 am and 12:00 pm, and total RNA was purified from peripheral leukocytes using the QIAamp RNA Blood Mini Kit (Qiagen, Chatsworth, California) according to the manufacturer’s manual. RNA quantity and quality were determined using a Nanodrop ND-1000 spectrophotometer (Thermo Fisher Scientific Inc.) and an Agilent Bioanalyzer (Agilent Technologies). The optical density (O.D.) 260/280 ratios were ≥1.5, and the RNA integrity numbers (RIN) were ≥7.0 for all RNA preparations.

### RNA isolation from mouse blood and brain tissue

All experimental protocols were approved by the Ethics Committee for Animal Experimentation of Yamaguchi University School of Medicine, and all experimental procedures were performed according to the Guidelines for Animal Care and Use Committee of the Yamaguchi University Graduate School of Medicine. Eight-week-old adult, male BALB/c mice (Charles River Laboratories Japan) were maintained on a 12-hour/12-hour light/dark cycle with ad libitum access to mouse chow and water.

In the first set of experiments, 17 mice were divided into the following three groups: non-stressed (N = 6), CUMS (N = 6), and CUMS + imipramine (IMI) (N = 5). The CUMS procedure, which is based solely on environmental and social stressors and does not include food/water deprivation (as previously described^13^), was performed using three stressors. For the first stressor, two of the following five ultra-mild diurnal stressors were delivered randomly over 1–2 hours with a 2-hour stress-free period between the two stressors: a period of cage tilt (30°), confinement to a small cage (11 × 8 × 8 cm), paired housing, soiled cage (50 ml water per 1 l of sawdust bedding), and odor (10% acetic acid). The second stressor consisted of four ultra-mild nocturnal stressors, which included one overnight period where mice had difficulty accessing their food, one overnight period with permanent light, one overnight period with a 30° cage tilt, and one overnight period where mice were confined to a soiled cage. For the third stressor, a reversed light/dark cycle was used from Friday evening to Monday morning. This procedure was scheduled over a 1-week period and was repeated six times. IMI, which was administered for the last 3 weeks during each CUMS session, was purchased from Sigma-Aldrich and was dissolved in tap water (with protection from light) at a concentration of 160 mg/l. Total RNA extraction was performed as follows. Mice from each group were anesthetized and sacrificed to obtain blood from their trunks (500–1000 µl), which was collected into heparinized tubes. Total RNA was extracted using the GeneJet Whole Blood RNA Purification Mini Kit (Thermo Fisher Scientific Inc.) according to the manufacturer’s instructions. RNA quantity and quality were determined using a Nanodrop ND-1000 spectrophotometer (Thermo Fisher Scientific Inc.) and an Agilent Bioanalyzer (Agilent Technologies). The O.D. 260/280 ratios were ≥1.5, and the RINs were ≥7.0 for all RNA preparations.

In the second set of experiments, 24 mice were divided into the following three groups: non-stressed, CUMS, and CUMS + IMI (N = 8 per group). Total RNA from the hippocampus and PFC of the sacrificed mice was extracted after CUMS using TRIzol Reagent (Invitrogen). The samples were treated with DNase (DNA-free; Ambion). The O.D. 260/280 ratios were ≥1.5 for all RNA preparations.

### Microarrays on human and mouse blood samples

We used all human leukocyte samples and 12 mouse blood samples obtained from four randomly selected mice from each group in the first microarray set.

Total RNA was amplified and labeled using Cyanine 3 (Cy3) using the Agilent Low Input Quick Amp Labeling Kit (Agilent Technologies, Palo Alto, CA) according to the manufacturer’s instructions. Briefly, 100 ng of total RNA were reverse transcribed to double-stranded cDNA using a poly dT-T7 promoter primer. The primer, template RNA, and quality-control transcripts of known concentration and quality were first denatured at 65 °C for 10 minutes and incubated for 2 hours at 40 °C with 5X first strand buffer, 0.1 M dithiothreitol, 10 mM deoxynucleotide mix, and AffinityScript RNase Block Mix. The AffinityScript enzyme was inactivated at 70 °C for 15 minutes. cDNA products were then used as templates for *in vitro* transcription to generate fluorescent cRNA. cDNA products were mixed with a transcription master mix in the presence of T7 RNA polymerase and Cy3-labeled cytidine triphosphate, and incubated at 40 °C for 2 hours. Labeled cRNAs were purified using QIAGEN’s RNeasy mini spin columns and eluted in 30 μl of nuclease-free water. After amplification and labeling, cRNA quantity and cyanine incorporation were determined using a Nanodrop ND-1000 spectrophotometer and an Agilent Bioanalyzer.

For each hybridization, 0.60 μg of Cy3-labeled cRNA was fragmented and hybridized at 65 °C for 7 hours using an Agilent SurePrint G3 Human GE v2 8 × 60 K Microarray (Design ID: 039494) or an Agilent SurePrint G3 Mouse GE 8 × 60 K Microarray (Design ID: 028005). After washing, the microarrays were scanned using an Agilent DNA microarray scanner.

### Microarray analysis

Intensity values of each scanned feature were quantified using Agilent feature extraction software version 10.7.3.1, which performed background subtractions. We only used features that were flagged as having no errors (Detected flags) and excluded features that were not positive, not significant, not uniform, not above background, or saturated, and those that were population outliers (Not Detected and Compromised flags). The normalization (per chip: 75th percentile shift) and statistical analysis of microarray data were performed using Agilent GeneSpring GX software version 11.0.2 (human) or Agilent GeneSpring GX version 13.0 (mouse). Without control probes being factored in, the Agilent SurePrint G3 Human GE v2 8 × 60 K Microarray (Design ID: 039494) contained a total of 50,599 probes, while the Agilent SurePrint G3 Mouse GE 8 × 60 K Microarray (Design ID: 028005) contained 55,681 probes.

For human data, probes were filtered based on signal intensity. Probes with raw signal intensity ≥50 in at least two of the samples were used. The final number of probes for subsequent analysis was 30,782. Differentially expressed genes between groups were identified using a one-way ANOVA followed by Tukey’s post hoc test. P-values were corrected using FDR (Benjamini and Hochberg adjustment method). The cutoff for the corrected p-value was 0.05.

For mouse data, probes were filtered based on flag calls defined using GeneSpring software. Probes labeled as “Detected” in at least 75 percent of samples in any one out of the three conditions were used. The final number of probes for subsequent analysis was 23,869. Differentially expressed genes between groups were identified using a one-way ANOVA followed by Tukey’s post hoc test. The cutoff for significance was a p-value of 0.05.

### Quantitative real-time polymerase chain reaction

cDNA synthesis and real-time Q-PCR were performed as previously described^9^. The list of primer sequences is shown in Table S3. All measurements were performed in duplicate. Levels of glyceraldehyde 3-phosphate dehydrogenase mRNA were used to normalize the relative expression levels of target mRNA, according to the methods used in previous reports^8–12^.

### Statistical analysis

EZR software was used for data analysis^59^. As age in the MDD-RM group was not normally distributed, participant ages in Experiment 1 were analyzed using a non-parametric test (Kruskal-Wallis test) with a Steel-Dwass post hoc analysis. For Experiment 2, the distribution of participant ages was determined using an unpaired Student’s t-test while the distribution of participant sexes was analyzed by Fisher’s Exact Test. For real-time Q-PCR data obtained in Experiment 1, the variance in expression of each candidate gene was not equal among the HC, MDD-DP, and MDD-RM groups, as determined by Bartlett’s test. Significant differences were determined using a non-parametric test (Kruskal-Wallis) with a Steel-Dwass post hoc analysis. For samples collected in Experiment 2, homogeneity of variances was determined using an F-test, and significant differences between the HC and MDD-DP groups were determined using an unpaired Student’s t-test or Welch’s t-test. For murine samples, significant differences were determined using a one-way ANOVA followed by a Tukey post hoc analysis. The Pearson coefficient was used to measure the correlations between mRNA expression levels and participant ages (including HC participants), disease durations, and antidepressant doses (excluding HC participants). A receiver operating characteristic (ROC) analysis was carried out using EZR. All data are expressed as the mean ± standard deviation (SD) or standard error of the mean (SEM).

## Electronic supplementary material

Dataset 1
