# Supplementary material for: Identification of commonly altered genes between in major depressive disorder and a mouse model of depression
Source: Sci Rep. 2017 Jun 8;7:3044. doi: 10.1038/s41598-017-03291-x (PMC5465183; doi:10.1038/s41598-017-03291-x)
Supplement: Supplementary file 1 — Dataset 1 [file 41598_2017_3291_MOESM1_ESM.doc]

Identification of commonly altered genes between in major depressive disorder and a mouse model of depression

Hirotaka Yamagata^1^*, Shusaku Uchida^1^, Koji Matsuo^1^, Kenichiro Harada^1^, Ayumi Kobayashi^1^, Mami Nakashima^1,2^, Masayuki Nakano^1,3^, Koji Otsuki^1,4^, Naoko Abe-Higuchi^1^, Fumihiro Higuchi^1^, Toshio Watanuki^1^, Toshio Matsubara^5^, Shigeo Miyata^6^,Masato Fukuda^6^, Masahiko Mikuni^6,7,8^, and Yoshifumi Watanabe^1^

1. Division of Neuropsychiatry, Department of Neuroscience, Yamaguchi University Graduate School of Medicine, 1-1-1 Minami-kogushi, Ube, Yamaguchi 755-8505, Japan

2. Nagatoichinomiya Hospital, 17-35 Katachiyama-midoricho, Shimonoseki, Yamaguchi, 751-0885 Japan

3. Katakura Hospital, 229-3 Nishikiwa, Ube, Yamaguchi 755-0151, Japan

4. Department of Psychiatry, Shimane University Faculty of Medicine, 89-1 Enya-cho, Izumo, Shimane, 693-8501, Japan.

5. Health Service Center Organization for University Education, Yamaguchi University, 1677-1 Yoshida, Yamaguchi-shi, Yamaguchi, 753-8511, Japan.

6. Departments of Psychiatry and Neuroscience, Gunma University Graduate School of Medicine, 3-39-22 Showa-machi, Maebashi, Gunma 371-8511, Japan.

7. Hakodate Watanabe Hospital, 1-31-1 Yunokawa-cho, Hakodate, Hokkaido, 042-8678, Japan.

8. Department of Psychiatry, Hokkaido University Graduate School of Medicine, North 15, West 7, Kita-Ku, Sapporo, Hokkaido, 060-8638, Japan

*Correspondence to: Hirotaka Yamagata, M.D., Ph.D.

Division of Neuropsychiatry, Department of Neuroscience, Yamaguchi University Graduate School of Medicine, 1-1-1 Minami-kogushi, Ube, Yamaguchi 755-8505, Japan

Phone: +81 836 22 2255; FAX: +81 836 22 2253

E-mail: gata@yamaguchi-u.ac.jp

Table S1. Top 50 significantly altered genes in leukocytes from patients with MDD in the depressed state (MDD-DP) compared to healthy controls (HCs).

Table S2. Top 50 significantly altered genes in blood cells from chronic, ultra-mildly stressed (CUMS) mice compared to non-stressed (NS) mice.

| Gene Symbol | Forward or Reverse | sequence(5' to 3') |
| --- | --- | --- |
| Slc35a3 | Forward | GGATTTGCGACCTCCTTATCC |
| Slc35a3 | Reverse | AGCTGCTATTACAAGGATGGC |
| Plpp5 | Forward | TCTGTGCCTTTCTGTCACCT |
| Plpp5 | Reverse | AGGCAAATGTCATTCCAATCATG |
| Erlin2 | Forward | TAACCAGTTCTGCAGCGTTC |
| Erlin2 | Reverse | AAGTCAGGTCCTGCTGCAAA |
| Ppfia1 | Forward | CGCTTAGACAGGATGCACAA |
| Ppfia1 | Reverse | TTACTGCTGCTAGGGTTGCT |
| Yeats4 | Forward | GCGGAATTTGGACCTGACTC |
| Yeats4 | Reverse | GTGCCCGTCTTCTTCTCTCT |
| Gapdh | Forward | AGGTCGGTGTGAACGGATTTG |
| Gapdh | Reverse | TGTAGACCATGTAGTTGAGGTCA |
| SLC35A3 | Forward | CAATCCAGTTGCTCCTGTGC |
| SLC35A3 | Reverse | ATGCAATGAAGAGATTAAGGGCT |
| PLPP5 | Forward | GAACACCGTGGGCATAAACA |
| PLPP5 | Reverse | TGCTGAAACCACACTCCTGA |
| ERLIN2 | Forward | ATCCACCACGAACTGAACCA |
| ERLIN2 | Reverse | GTCCTGTTGCAAAGCCAGTT |
| HIST1H2AL | Forward | AGAAGACCCGCATTATCCCG |
| HIST1H2AL | Reverse | TCGGTCTTCTTGGGCAGTAG |
| PPFIA1 | Forward | GGACATTCGTGGCTTAGCTG |
| PPFIA1 | Reverse | TCCAACCTCTGTGTTCCTGA |
| YEATS4 | Forward | AGATTGCAGAGCTTAAGGAGAGA |
| YEATS4 | Reverse | TGCTTGGTCATCTTCTTCAAGT |
| GAPDH | Forward | CAGCCTCAAGATCATCAGCA |
| GAPDH | Reverse | TGTGGTCATGAGTCCTTCCA |

Table S3. List of primer sequences.


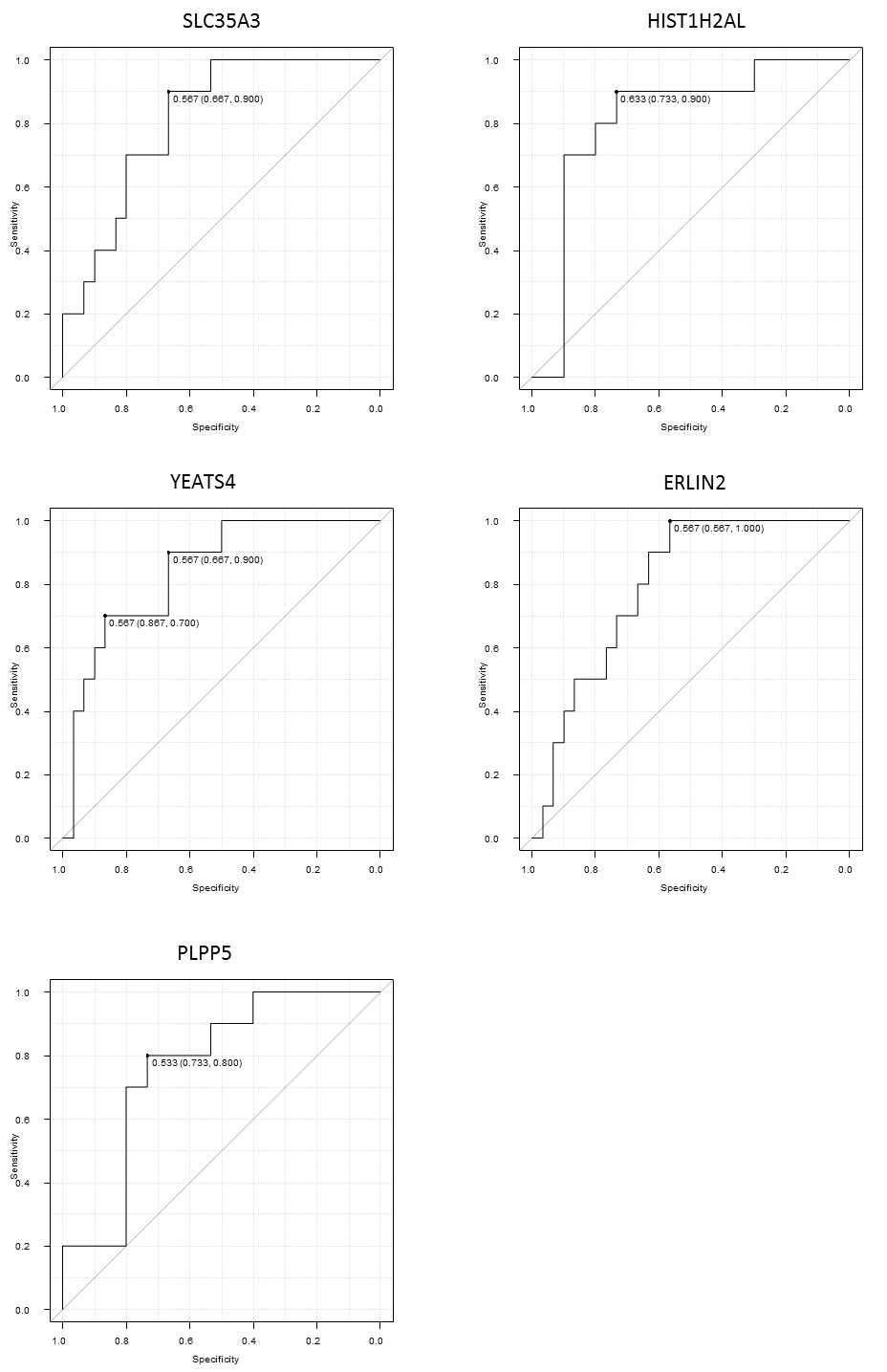


Figure S1. ROC curves for the expression levels of SLC35A3, HIST1H2AL, YEATS4, ERLIN2, and PLPP5 in participants from Experiment 1. The maximum values of Youden’s index (sensitivity and specificity) are represented on the ROC curve.


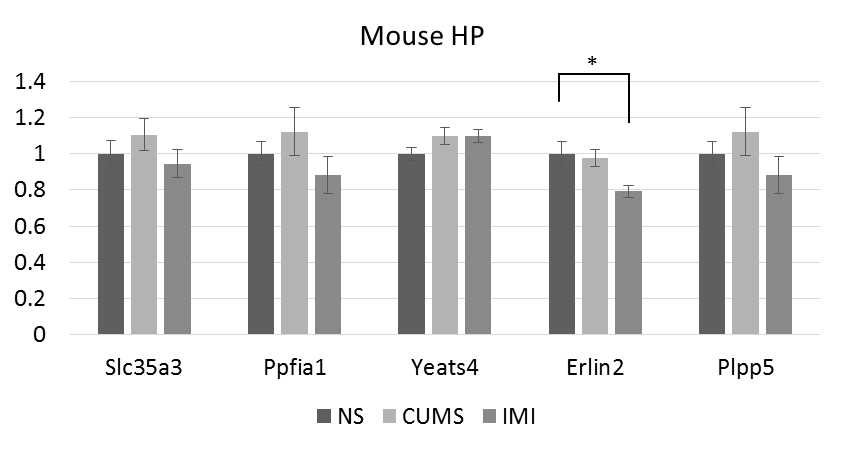


Figure S2. Expression levels of candidate genes in the murine hippocampus (HP)

Expression levels of Slc35a3, Ppfia1, Yeats4, Erlin2, and Plpp5 mRNA from mice the NS, CUMS, and IMI groups (NS, N = 8; CUMS, N = 8; IMI, N = 8). Data indicate the mean ± SEM. * p < 0.05.
